# Advancing Cancer Immunotherapy: From Molecular Mechanisms to Clinical Applications

**DOI:** 10.3390/cancers15164197

**Published:** 2023-08-21

**Authors:** Xianda Zhao, Timothy Starr, Subbaya Subramanian

**Affiliations:** 1 Department of Surgery, University of Minnesota Medical School, Minneapolis, MN 55455, USA; zhaox714@umn.edu; 2 Department of Obstetrics, Gynecology and Women’s Health, Masonic Cancer Center, University of Minnesota, Minneapolis, MN 55455, USA; star0044@umn.edu; 3 Center for Immunology, University of Minnesota, Minneapolis, MN 55455, USA

In recent years, cancer immunotherapy research has made remarkable progress, completely transforming the cancer treatment landscape. In 2021, we proudly introduced the Special Issue “Cancer Immunology” in the journal *Cancers*, featuring a collection of 17 highly acclaimed research and review articles [1]. Building upon the success of this prior endeavor, we are excited to present this new Special Issue, which aims to delve deeper into the most recent advancements, state-of-the-art technologies, and prospects in fundamental cancer immunology, pre-clinical assessments, and clinical trials.

Increased understanding of the molecular mechanisms governing the anti-tumor immune response has led to a surge in the utilization of innovative cancer immunotherapies across various cancer types. However, it is important to acknowledge that the response to cancer immunotherapies remains limited to a small subset of patients with solid tumors and specific hematopoietic malignancies, with the underlying reasons for its failure in other patients largely unidentified [2,3]. In this Special Issue, we have collected seven meticulously conducted original studies and five expertly written review articles, highlighting the unmet challenges and needs of cancer immunotherapies.

Over the past decade, the blockade of programmed cell death protein 1 (PD-1), programmed death-ligand 1 (PD-L1), and cytotoxic T-lymphocyte–associated antigen 4 (CTLA-4) has emerged as a cornerstone of cancer immunotherapy. Our initial series of articles focuses on the latest advancements in immune checkpoint inhibitors. Classical Hodgkin lymphomas, particularly in cases of relapse or refractory diseases, pose a significant treatment challenge. In their phase 2 clinical trial, Hanel et al. [4] explored the combination of nivolumab with Bruton’s tyrosine kinase inhibitor ibrutinib and observed durable responses, even among patients who had previously progressed on nivolumab therapy. In another study, Pi et al. [5] demonstrated a correlation between COX2 expression and resistance to anti-PD-1 therapy in a B16F10 animal model. Notably, inhibiting COX2 or knocking out the Ptgs2 gene in the B16F10 model reversed its resistance to anti-PD-1 treatment. These noteworthy findings provide valuable insights into potential strategies to overcome immune checkpoint inhibitor resistance.

Chimeric antigen receptor (CAR) T cell therapy represents another vital branch of tumor immunotherapy. Liang et al. [6] made an important discovery, demonstrating that the pharmacological facilitation of CAR-induced autophagy with verteporfin can effectively inhibit the trogocytic expression of tumor antigens on CARs. This breakthrough finding not only enhances CAR persistence and efficacy in mice, but also holds the potential to extend the duration of CAR T cell therapy in patients.

Conventional cancer treatments, such as chemotherapy, radiation therapy, and inhibitors targeting oncogenic pathways, have demonstrated remarkable immunoregulatory effects within tumor tissues [7]. Monoclonal antibodies designed to target oncogenic pathways can activate antibody-dependent cell-mediated cytotoxicity (ADCC), thereby eliminating cancer cells through immunological mechanisms. Troschke-Meurer et al. [8] revealed that combining chemotherapeutics with Dinutuximab beta in the presence of immune cells significantly enhanced their cytotoxic efficacy against neuroblastoma, with the effect specific to the GD2 antigen. Additionally, the same group reported that the immunocytokine, FAP-IL-2v, in combination with the anti-GD2 antibody Dinutuximab beta, substantially increased ADCC against neuroblastoma cells. This synergistic approach involving Dinutuximab beta and FAP-IL-2v resulted in a significant reduction in tumor growth and improved survival in experimental mice [9].

Two insightful studies conducted by Zou et al. [10] and Drachneris et al. [11] have delved into potential biomarkers associated with the response to cancer immunotherapy. In their research, Drachneris et al. [11] examined a cohort of 157 high-risk, non-muscle invasive papillary urothelial carcinoma patients who underwent Bacille Calmette–Guerin immunotherapy following a transurethral resection. They discovered that gradient indicators of CD8^+^ cell densities at the tumor epithelium-stroma interface, in conjunction with routine clinical and pathology data, significantly enhanced the prediction of recurrence-free survival. On the other hand, in the context of pancreatic adenocarcinoma, Zou et al. [10] established a correlation between estrogen receptor expression and the development of tertiary lymphoid structures, indicating that estrogen receptors may potentially contribute to anti-tumor immune responses.

In addition to the original articles presented, this Special Issue includes a selection of timely review articles that provide comprehensive insights into the latest advancements. Fanciulli et al. [12] conducted a meticulous analysis of pre-clinical and clinical data concerning CAR T cell therapy in neuroendocrine neoplasms, highlighting its promising potential in clinical practice. Choudhary et al. [13] discussed the metabolic alterations observed in glioblastoma tumor cells, which have been investigated as contributing factors to immunosuppression and resistance against immunotherapies. Monteleone et al. [14] reviewed the current body of evidence supporting the role of IL-34 in the differentiation and function of immune suppressive cells. Frak et al. [15] and Wei et al. [16] summarized recent progress in understanding how bacteria can influence the immune response against cancer, and their potential as a novel avenue for cancer immunotherapy. In addition, Gerton et al., showed epigenetic reprogramming and patient-derived 3D platforms could also be used to enhance immunotherapeutic responses in high-grade serous ovarian cancer [17].

In recent years, cancer immunotherapy has made remarkable progress, transforming cancer treatment. The Special Issue “Tumor Immunology and Immunotherapy Resistance” highlighted breakthroughs, technologies, and prospects in cancer immunology. While immune checkpoint inhibitors have been revolutionary, addressing limited responses is crucial. Studies in this Special Issue suggest strategies to overcome resistance, including combination therapies and specific pathway targeting.

Chimeric antigen receptor (CAR) T cell therapy is an important branch of immunotherapy. Facilitating CAR-induced autophagy has shown promise in enhancing CAR T cell therapy’s effectiveness. Conventional treatments and monoclonal antibodies can also activate immunological mechanisms, offering synergistic approaches to enhance cytotoxic efficacy against specific cancers. Moreover, identifying biomarkers associated with treatment response is essential for personalized cancer immunotherapy. Studies in this Special Issue discuss potential biomarkers, such as immune cell densities and estrogen receptor expression, enabling more effective interventions. Notably, the review articles cover advancements in CAR T cell therapy, metabolic alterations in tumors, the role of immune suppressive cells, and the influence of bacteria on the immune response against cancer.

As we embark on the next generation of cancer immunotherapy, an increasing body of work has demonstrated that tumor-derived extracellular vesicles are key immune modulators in tumor signaling and the determinants of the antitumor immune response [18]. This Special Issue is an invaluable resource for researchers, clinicians, and industry professionals. The knowledge and discoveries presented within these articles will guide future developments, bringing us closer to realizing more effective and personalized cancer immunotherapies.
